# Effect of Selenium Application and Growth Stage at Harvest on Hydrophilic and Lipophilic Antioxidants in Lamb’s Lettuce (*Valerianella locusta* L. Laterr.)

**DOI:** 10.3390/plants10122733

**Published:** 2021-12-12

**Authors:** Liubov Skrypnik, Tatiana Styran, Tamara Savina, Nadezhda Golubkina

**Affiliations:** 1 Institute of Living Systems, Immanuel Kant Baltic Federal University, 236040 Kaliningrad, Russia; TStyran@stud.kantiana.ru (T.S.); TSavina@stud.kantiana.ru (T.S.); 2 Analytical Laboratory Department, Federal Scientific Vegetable Center, 143072 Moscow, Russia; segolubkina45@gmail.com

**Keywords:** biofortification, leafy green vegetable, corn salad, field salad, phenolic compounds, chlorogenic acid, lutein

## Abstract

Lamb’s lettuce (*Valerianella locusta* L. Laterr.) is a leafy green vegetable that is rich in various biological active compounds and is widely used in ready-to-eat salads. The cultivation conditions and growth stage could affect the secondary metabolism in plants and thereby modify their food value. In the present study, the effect of selenium (Se) application in various concentrations (5.0, 10.0, and 20.0 µM) on the contents of Se, phenolic compounds, vitamin C, carotenoids, chlorophylls, and antioxidant activity of hydrophilic and lipophilic extracts of lamb’s lettuce harvested at three growth stages (38, 52, and 66 days after sowing (DAS)) was studied. Se application significantly increased the Se concentration in the shoots (up to 124.4 μg g^−1^ dry weight), as well as the contents of chlorogenic acid, total flavonoids, total phenolics, ascorbic acid, chlorophyll *b*, and the antioxidant activity of hydrophilic and lipophilic extracts. A higher content of phenolic compounds and higher antioxidant activity of hydrophilic extracts was observed at the first growth stage (38 DAS). On the contrary, higher contents of lipophilic compounds (chlorophyll *a*, chlorophyll *b*, lutein, β-carotene) and higher antioxidant activity of lipophilic extracts were found for shoots harvested at later stages (52 and 66 DAS).

## 1. Introduction

Lamb’s lettuce (*Valerianella locusta* L. Laterr.), also called corn salad or field salad, is a leafy green vegetable widely used in ready-to-eat salads. The nutritional value of lamb’s lettuce is due to its high content of carotenoids, phenolic compounds, folic acid, sterols, and fatty acids [1,2]. In addition, lamb’s lettuce is frost-resistant, so it can be grown in different regions, including regions with temperate climates [3]. Lamb’s lettuce cultivation in greenhouses is also widespread, including the hydroponic technique [4].

Among the main factors determining the phytochemical composition of leafy vegetables and their antioxidant properties, in addition to the genotype of plants, the stages of development of crops, and conditions of their cultivation are included. The study of these factors is of particular interest since by varying them, it is possible to maximize the content of phytonutrients beneficial to health [5]. The data available in the literature on changes in the level of antioxidant components in various leafy vegetables at various stages of growth are contradictory. For example, some studies have shown that plants at the early stages of growth were distinguished by a higher total content of phenolic compounds [6,7], while in others, opposite results were obtained [8,9,10]. As far as is known, there is no information about the effect of the growth stage on the antioxidant properties of lamb’s lettuce.

The cultivation conditions could affect the secondary metabolism in lamb’s lettuce plants and thereby modify their food value. Thus, it was shown that supplemental lighting of lamb’s lettuce plants using light-emitting diodes improved the biochemical composition of two cultivars of lamb’s lettuce, in particular, the contents of soluble sugars, ascorbic acid, and polyphenols and the antioxidant activity increased [11]. Mola et al. found that the content of ascorbic acid and phenolic compounds and the antioxidant activity in lamb’s lettuce was influenced by the dose of nitrogen fertilizers and foliar treatment with plant-based biostimulants [12].

Selenium biofortification of green vegetables could also lead to changes in the content of some biologically active compounds with antioxidant properties and it could also improve the nutrition value of vegetables due to Se being essential for humans. Consumption of selenium less than the recommended daily allowance (50–60 µg per day) can lead to the development of more than 40 different diseases; therefore, its use as a fertilizer for crops is necessary to increase its level in the human diet [13,14,15]. In plants, low and moderate concentrations of Se stimulate growth and development, as well as affecting their secondary metabolism. This provides an opportunity to use exogenous selenium to improve the nutritional value of plants [16,17]. It is known that selenium has a significant effect on the secondary metabolism of sulfur-containing compounds, in particular, glutathione, glucosinolates, and isothiocyanates [11,12]. However, recent studies have shown that the application of exogenous selenium also affects the concentration of phenolic compounds, ascorbic acid, and other antioxidants in plants [18,19].

Studies on selenium biofortification of lamb’s lettuce are very limited. Tomasi et al. (2015) investigated the effect of the introduction of exogenous selenium into the nutrient solution on yield, selenium accumulation, and the content of nitrates and sulfur- and selenium-containing amino acids in lamb’s lettuce [20]. Hawrylak-Nowak et al. (2018) studied the promotion effect of Se biofortification on lamb’s lettuce grown under high-temperature stress [21]. The purpose of this recent study was to study the effect of selenium application at various concentrations (5.0, 10.0, and 20.0 µM) on the contents of selenium, phenolic compounds, vitamin C, carotenoids, and chlorophylls, and the antioxidant activity of hydrophilic and lipophilic extracts of lamb’s lettuce harvested at three stages of growth (38, 52, and 66 days after sowing). According to the best of our knowledge, studies on the effect of selenium on the antioxidant properties of lamb’s lettuce at different growth stages have not been conducted before. The findings from the present study will add to our understanding of the effect of selenium biofortification on the nutritional quality of green leafy vegetables.

## 2. Results

### 2.1. Effect of Se Application and Growth Stage at Harvest on Plant Yield and Accumulation of Se

The control lamb’s lettuce plants were characterized by a low concentration of selenium in the shoots, which ranged from 0.011 to 0.051 µg g^−1^, depending on the stage of plant growth (Table 1). When 5 µM of selenium was added to the nutrient solution, its concentration in the lamb’s lettuce increased to 10.7 µg g^−1^. The maximum concentration of selenium (124.4 µg g^−1^) was observed in the shoots of lamb’s lettuce grown on a nutrient solution containing selenium at a concentration of 20 µM and harvested at 52 days after sowing (DAS).

**Figure 1:**
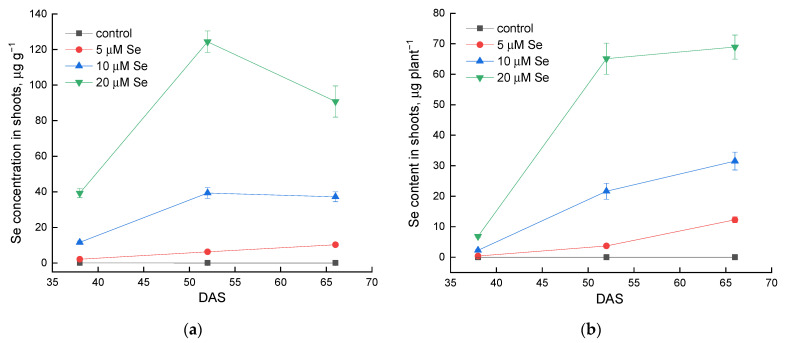
Selenium (Se) concentration (**a**) and Se content (**b**) in lamb’s lettuce shoots at different growth stages depending on the amount of selenium application. Data are presented as mean ± standard deviation (*n* = 4). DAS, days after sowing.

To study the dynamics of selenium accumulation during lamb’s lettuce growth and taking into account the process of dilution of its concentration in the plant due to an increase in plant biomass, the selenium content per plant (µg plant^−1^) was calculated. The data presented in Figure 1a (selenium concentration, µg g^−1^) demonstrate a sharp decrease in selenium concentration by 66 DAS (from 124.4 µg g^−1^ by 52 DAS to 90.8 µg g^−1^ by 66 DAS) when 20 µM of selenium was added to the nutrient solution. However, from Figure 1b (selenium content, µg plant^−1^), it can be seen that the selenium content has not significantly changed since 55 DAS.

The study of the effect of different selenium concentrations on the accumulation of biomass by lamb’s lettuce showed that there were no significant changes in the fresh and dry weight of the shoots at the first two growth stages (Table 1). At the last growth stage, the addition of 5 µM of selenium had a stimulating effect on the accumulation of fresh and dry weight. The addition of 20 µM led to a decrease in the fresh weight of the plant. There were no significant changes in the dry matter content when selenium was added (Table 1).

### 2.2. Effect of Se Application and Growth Stage at Harvest on the Content of Hydrophilic Antioxidative Compounds

**Figure 2:**
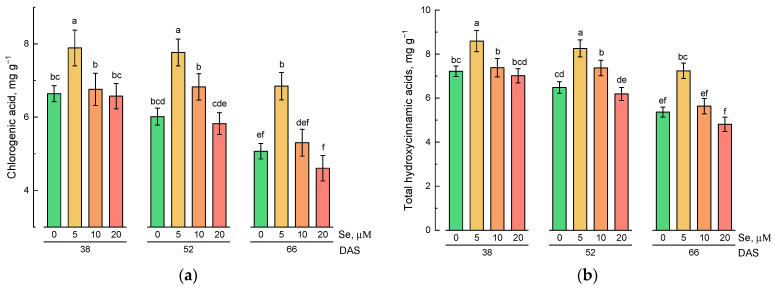

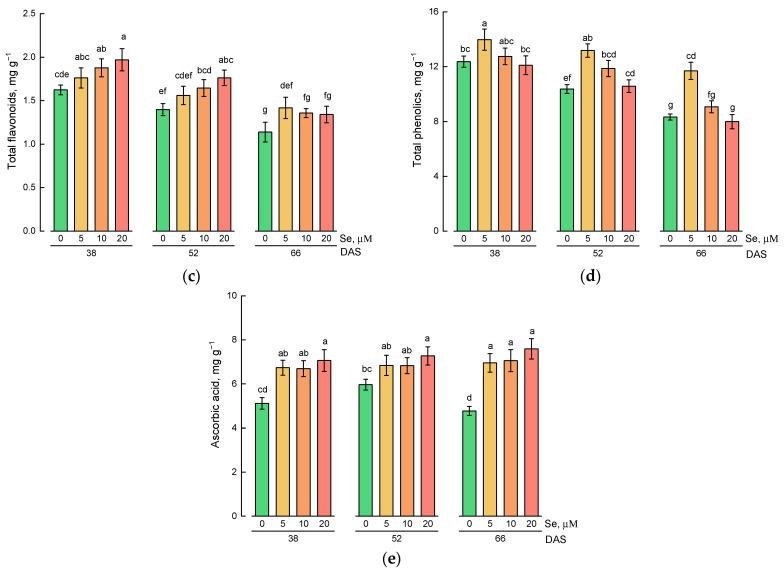
Effect of Se application and growth stage at harvest on the content of phenolic compounds and ascorbic acid: (**a**) chlorogenic acid; (**b**) total hydroxycinnamic acids; (**c**) total flavonoids; (**d**) total phenolics; (**e**) ascorbic acid. Data was evaluated via one-way ANOVA followed by the Tukey HSD test (mean ± SD, *n* = 4). Identical letters indicate that mean values do not differ significantly (*p* > 0.05).

Analysis of the content of chlorogenic acid and total hydroxycinnamic acids content in lamb’s lettuce shoots showed that the addition of 5 µM of Se to the nutrient solution stimulated the accumulation of these phytochemicals at all stages of growth compared with control plants (Figure 2a,b). At the same time, during plant growth, the average content of hydroxycinnamic acids decreased. The maximum contents of chlorogenic acid were observed at 38 and 52 DAS with the addition of 5 µM of Se and amounted to 7.89 and 7.76 mg g^−1^, respectively.

The total content of flavonoids in lamb’s lettuce increased with the increase in the selenium concentration in the nutrient solution at the first and second stages of harvesting (Figure 2c). At the third stage, a significantly higher level of flavonoids compared to the control was observed only with the addition of 5 µM of Se. A decrease in the accumulation of flavonoids with the growth of lamb’s lettuce was observed.

The total content of phenolic compounds was significantly higher compared to the control with the addition of 5 µM of Se at all stages of lamb’s lettuce growth (Figure 2d). With the growth of plants, as well as for hydroxycinnamic acids and flavonoids, a decrease in the total content of phenolic compounds was observed.

The addition of selenium to the nutrient solution led to a significant increase in the content of ascorbic acid in lamb’s lettuce shoots (Figure 2e). At the same time, no significant changes in the content of ascorbic acid during plant growth were observed.

**Figure 3:**
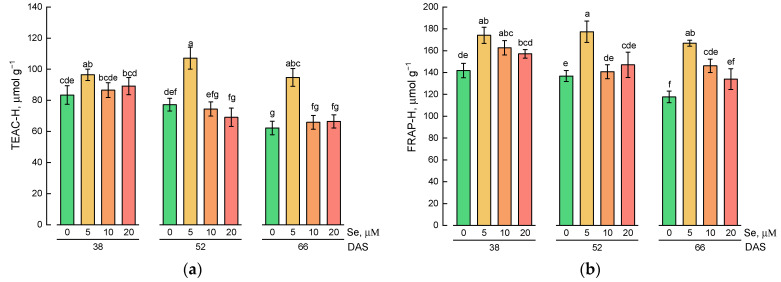
Effect of Se application and growth stage at harvest on (**a**) Trolox equivalent antioxidant capacity of hydrophilic extracts (TEAC-H) and (**b**) ferric-reducing antioxidant power of hydrophilic extracts (FRAP-H). Data was evaluated via one-way ANOVA followed by the Tukey HSD test (mean ± SD, *n* = 4). Identical letters indicate that mean values do not differ significantly (*p* > 0.05).

A study of the antioxidant activity of hydrophilic lamb’s lettuce extracts showed that the application of selenium at a concentration of 5 µM led to an increase in antioxidant activity compared with the control plants at all stages of growth (Figure 3a,b). The maximum antioxidant activity measured by Trolox equivalent antioxidant capacity (TEAC) and ferric-reducing antioxidant power (FRAP) assays was observed for extracts of lamb’s lettuce grown on nutrient solution containing 5 µM of selenium and harvested at 52 DAS.

Correlation analysis of data on the content of phenolic compounds and ascorbic acid, and the antioxidant activity of hydrophilic extracts showed that the greatest contribution to the antioxidant activity of lamb’s lettuce extracts was made by chlorogenic acid, the sum of hydroxycinnamic acids, and the sum of phenolic compounds (Table 2).

### 2.3. Effect of Se Application and Growth Stage at Harvest on Lipophilic Antioxidative Compounds

**Figure 4:**
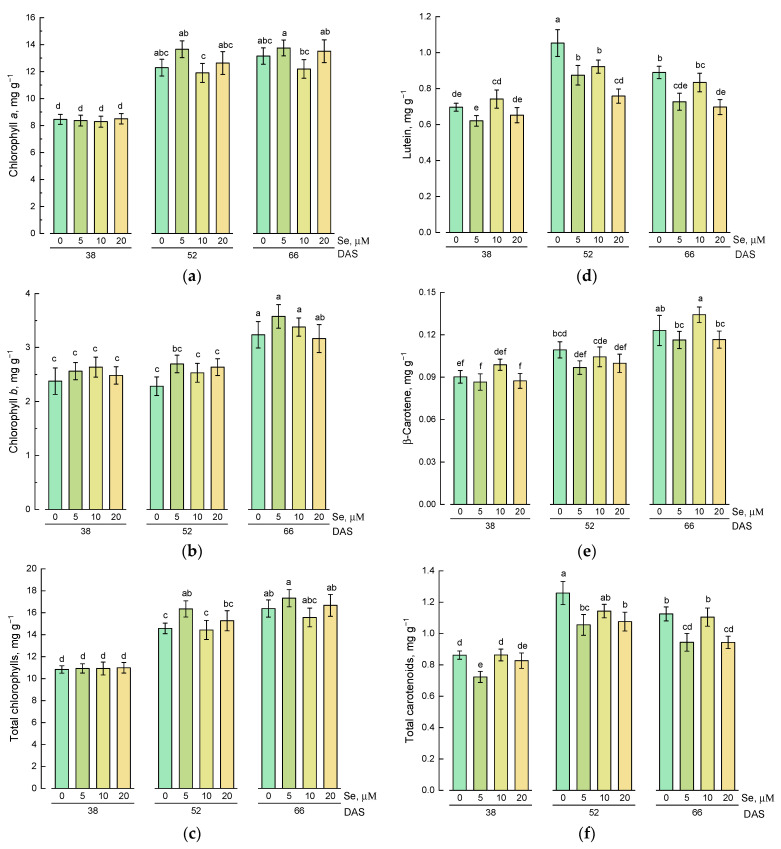
Effect of Se application and growth stage at harvest on the content of chlorophylls and carotenoids: (**a**) chlorophyll *a*; (**b**) chlorophyll *b*; (**c**) total chlorophylls; (**d**) lutein; (**e**) β-carotene; (**f**) total carotenoids. Data was evaluated via one-way ANOVA followed by the Tukey HSD test (mean ± SD, *n* = 4). Identical letters indicate that mean values do not differ significantly (*p* > 0.05).

The study of the effect of different concentrations of selenium in the nutrient solution on the content of chlorophyll *a* showed that at the first stage of growth (38 DAS), selenium did not have a significant effect on the content of this pigment (Figure 4a). At the second and third stages, the addition of 10 µM of selenium led to a decrease in the level of chlorophyll *a*. The content of chlorophyll *a* was significantly higher in plants harvested at 52 and 66 DAS compared with plants harvested at 38 DAS.

The content of chlorophyll *b* in the shoots of lamb’s lettuce harvested at 38 DAS also did not depend on the presence of selenium in the nutrient solution (Figure 4b). On average, the content of chlorophyll *b* was higher in plants harvested at the third stage of growth (66 DAS).

Since the content of chlorophyll *a* was about three times higher compared to chlorophyll *b*, the trend in the change in the total chlorophylls content was similar to the changes in the content of chlorophyll *a* (Figure 4c).

The study of the effect of different concentrations of selenium in the nutrient solution on the content of lutein and β-carotene in lamb’s lettuce showed that the addition of selenium did not have a stimulating effect on the accumulation of these carotenoids (Figure 4d,e). The content of lutein and β-carotene in plants grown on a substrate containing selenium was comparable to the control plants or even lower. The maximum lutein content and total content of carotenoids were found in control plants harvested at 52 DAS (Figure 4d,f). On average, plants harvested at 66 DAS had a higher content of β-carotene compared with younger plants (Figure 4e).

**Figure 5:**
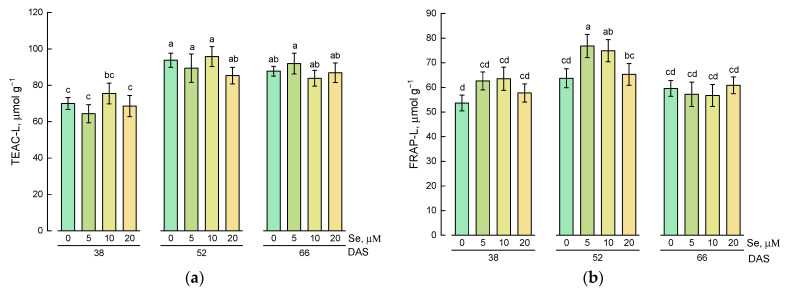
Effect of Se application and growth stage at harvest on (**a**) Trolox equivalent antioxidant capacity of lipophilic extracts (TEAC-L) and (**b**) ferric-reducing antioxidant power of lipophilic extracts (FRAP-L). Data was evaluated via one-way ANOVA followed by the Tukey HSD test (mean ± SD, *n* = 4). Identical letters indicate that mean values do not differ significantly (*p* > 0.05).

The TEAC of lipophilic extracts of lamb’s lettuce treated with exogenous selenium did not differ from the values obtained for control plants at all stages of growth (Figure 5a). Plants harvested at 52 and 66 DAS were characterized by higher antioxidant activity compared with plants harvested at 38 DAS.

The study of FRAP of lipophilic extracts showed that higher levels of this parameter were typical for plants grown on a nutrient solution with the addition of 5 and 10 µM of selenium (Figure 5b).

Correlation analysis of data on the content of chlorophylls and carotenoids, and the antioxidant activity of lipophilic extracts showed that the greatest contribution to the antioxidant activity of lamb’s lettuce, determined by the TEAC assay, was made by chlorophyll *a*, the sum of chlorophylls, lutein, and the sum of carotenoids (correlation coefficients were 0.82, 0.80, 0.74, and 0.80, respectively) (Table 3). The correlation coefficients between the antioxidant activity determined by the FRAP assay and the content of chlorophylls and carotenoids were lower. A significant moderate correlation was established between the antioxidant activity and the contents of chlorophyll *a* (*r* = 0.30), lutein (*r* = 0.44), and the sum of carotenoids (*r* = 0.35).

## 3. Discussion

### 3.1. Effect of Exogenous Se Application on Se Accumulation in Lamb’s Lettuce Shoots and Plant Yield

According to the ability to accumulate selenium, plants can be divided into three groups: non-accumulators, accumulators, and hyperaccumulators [22]. Plants of the first group accumulate up to 100 mg Se kg^−1^ DW, the second from 100 to 1000 mg Se kg^−1^ DW, and the third from 1000 to 15,000 mg Se kg^−1^ DW without symptoms of selenium toxicity. The selenium concentration in the control lamb’s lettuce plants in our experiment ranged from 0.011 to 0.051 µg g^−1^ (i.g. 0.011–0.051 mg Se kg^−1^). The maximum concentration of selenium was 124.4 µg g^−1^ (i.g. 124.4 mg Se kg^−1^). At this concentration of selenium in plant tissue, there was no significant decrease in the fresh and dry biomass of lamb’s lettuce compared to control plants (Table 1). The negative effect of selenium on lamb’s lettuce growth was established only for plants grown for a long time on the nutrient solution containing the maximum studied concentration of selenium (20 µM) and harvested at 66 DAS. In the previously study by Tomasi et al. (2015) on selenium biofortification of two cultivars of lamb’s lettuce, it was found that when plants were grown for 45 days on a solution containing 40 µM of selenium, plants accumulated from 77.4 to 96.4 mg Se kg^−1^ without significant changes in the leaf yield [20]. As shown by our two-way ANOVA, a low concentration of selenium (5 µM) had a stimulating effect on the accumulation of fresh lamb’s lettuce biomass (Table 1). The result of the positive effect of low concentrations of selenium on the growth of lamb’s lettuce is consistent with previously published results for the study of different species of plants, such as spinach [23], lettuce [24], Indian mustard [25], and faba bean [26]. The phenomenon that a low concentration of selenium stimulates plant growth whereas a high concentration of selenium has a toxic effect is regarded as hormesis and was proven for selenium in many previous studies [27,28]. The toxic effect of high concentrations of selenium is well studied and is usually associated with the inclusion of selenium instead of sulfur in amino acids and peptides and with the induction of oxidative and nitrosative stresses by selenium, which leads to metabolic disorders and damage to cellular structures [29]. The stimulating effect of low selenium concentrations on plant growth may be due to a positive effect on the distribution and assimilation of important nutrients, improvement of some physiological processes, in particular, an increase in the rate of photosynthesis, stomatal conductivity, and transpiration rate, as well as the antioxidant properties of selenium and its organic compounds and its ability to increase the activity of some antioxidant defense enzymes [29]. In addition, some authors do not exclude the connection of the stimulating effect of selenium with its effect on the hormonal balance and the level of polyamines in plants [23].

A study of the dynamics of selenium accumulation during lamb’s lettuce growth showed that when using a high concentration of selenium (20 µM), plants actively absorbed selenium from the nutrient solution during the first 4 weeks of the experiment. A similar dependence of selenium accumulation in plants on its concentration and the duration of growing plants on a solution containing selenium was established for basil and other vegetables in previous studies [30]. Thus, if the goal is to obtain lamb’s lettuce plants with a maximum concentration of selenium, then the best harvest time is at 52 DAS after 4 weeks of selenium treatment at a concentration of 20 µM.

However, when choosing the concentration of exogenous selenium for the biofortification of lamb’s lettuce, it should be borne in mind that high doses of selenium are toxic to humans. Recommended dietary allowances of selenium are on average 55–70 µg Se day^−1^, and the toxic threshold is 400 µg day^−1^ [14]. If one recalculates our results obtained for the concentration of selenium in lamb’s lettuce on fresh mass and takes into account that the average portion of salad is 50 g, then the maximum intake of selenium into the human body will be 43 µg when using 5 µM, 160 µg when using 10 µM, and 496 µg when using 20 µM. Thus, the use of selenium at a concentration of 20 µM, or even at a concentration of 10 µM, can potentially lead to the threshold level set for selenium consumption being exceeded. Thus, based on the obtained results of stimulating the accumulation of fresh biomass and the accumulation of selenium in plant tissue, it seems more appropriate and safe to recommend selenium application at a concentration of 5 µM if it is planned to be produced for sale in the form of ready-to-eat salads.

### 3.2. Effect of Exogenous Se Application on the Content of Hydrophilic and Lipophilic Antioxidants in Lamb’s Lettuce Shoots

It is known that biofortification with selenium leads not only to an increase in the content of this trace element in plants but can also affect the level of some secondary metabolites, including those with a nutritional value in the human diet [16,17]. For example, it was shown that the addition of selenium contributed to an increase in the content of phenolic compounds in basil [31,32,33], lettuce [34,35], spinach [23], coriander, and tatsoi microgreens [36]. In our study, the maximum contents of chlorogenic acid, the total hydroxycinnamic acids, and the total phenolic compounds were found at selenium application at a concentration of 5 µM (Appendix A, Table A1). Earlier, some authors noted that the dependence of the accumulation of phenolic compounds on the concentration of selenium had a U-shaped relationship, in which only at optimal concentrations of exogenous selenium was an increase in the content of phenolic compounds observed [33,37]. It is worth noting that the exception to this rule in our study was the content of flavonoids, the maximum of which was observed at the highest concentration of selenium. Unfortunately, the mechanisms explaining the effect of selenium on the biosynthesis of phenolic secondary metabolites have not been identified yet. Even though some studies have shown that the use of exogenous selenium in some plant species led to an increase in the expression of some genes and the activity of enzymes involved in the phenylpropanoid biosynthetic pathway [34,38,39,40], it remains unclear through which signal pathways and cellular response regulators selenium mediates the upregulation of these genes and/or affects the activity of enzymes.

The use of exogenous selenium also led to a significant increase in the content of ascorbic acid compared to control plants (Appendix A, Table A1). Although, as with phenolic compounds, additional studies are required to explain the mechanism of selenium’s effect on ascorbic acid biosynthesis, the experimental material accumulated to date confirms the possibility of increasing the content of ascorbic acid in various types of leafy green vegetables when using selenium [25,41,42]. The importance of increasing the ascorbic acid content in green leafy vegetables is also because an increased vitamin C content can improve the bioavailability of Fe and Zn contained in plant foods, and thus selenium biofortification additionally affects the synergy of nutrients [43].

Chlorophylls are basic photosynthesis pigments of plants and important as phytonutrients. It is known that chlorophylls possess antioxidative, anticarcinogenic, and antimutagenic properties and are capable of preventing the development of neurodegenerative diseases [44]. Our study did not reveal a significant stimulating effect of selenium on the accumulation of chlorophyll *a*. The accumulation of chlorophyll *b* was stimulated by the application of 5 and 10 µM of selenium into the nutrient solution; the total chlorophylls content was significantly higher only at 5 µM of selenium (Appendix A, Table A2). At the same time, the increase in the content of chlorophylls in comparison with the control did not exceed 10%. The data available in the literature on the effect of selenium on the accumulation of chlorophylls in green leafy vegetables are contradictory. For example, studies on spinach have shown that the use of low selenium concentrations contributed to an increase in the contents of chlorophyll *a*, chlorophyll *b*, and total chlorophylls [23]. While in the work of Malorgio et al., it was found that selenium treatment did not affect the content of chlorophylls in lettuce and chicory leaves [45].

This study of the effect of different selenium concentrations on the contents of lutein, β-carotene, and total carotenoids showed that selenium did not have a stimulating effect on the accumulation of these pigments. Only at a selenium concentration of 10 µM, the carotenoid content was comparable to the control plants, while at other concentrations, the pigment level was lower (Appendix A, Table A2). A decrease in carotenoids with the use of selenium was also found in the study of [35,46]. There were no significant changes in the carotenoid content in basil leaves [32] and kale [47] by selenium application. The decrease in the carotenoid content when using selenium may be due to the downregulation of phytoene synthase, a key enzyme in carotenoid biosynthesis, established on a model *Arabidopsis* plant grown by the addition of sodium selenate to the nutrient solution [48]. However, such genetic regulation and the corresponding changes in the content of carotenoids when using exogenous selenium strongly depend on the genotype of the plant.

The total antioxidant activity of plant extracts is attributed to three different types of interaction, namely the synergistic effect, antagonistic effect, and additive effect [49]. For example, a synergistic increase of the antioxidant activity was reported between caffeic acid and resveratrol, rosmarinic acid and quercetin, and α-tocopherol and ascorbic acid; antagonistic effect was observed in the mixtures of ascorbic acid and quercetin, and α-tocopherol and caffeic acid. For the mixtures of chlorogenic acid and rutin, chlorogenic acid and quercetin 3-glucoside, and catechin and resveratrol, the additive effect was observed [50]. In our study, the effect of the selenium application on the antioxidant activity of hydrophilic and lipophilic extracts of lamb’s lettuce, in general, reflected the trend in the changes in the content of individual phytochemicals (Appendix A, Table A1 and Table A2). This result confirms the assumption that the effect of selenium on antioxidant activity is most likely due to its effect on the accumulation of secondary metabolites with antioxidant properties, rather than the direct antioxidant effect of selenium itself and its organic metabolites.

The hydrophilic antioxidant components of lamb’s lettuce were more sensitive to the selenium application than lipophilic. This result may be related, among other things, to the fact that selenium enters and is metabolized in plants in the form of water-soluble compounds, such as selenate ion, selenocysteine, selenomethionine, methyl-selenocysteine, selenomethyl-selenocysteine, and γ-glutamyl-Se-methylselenocysteine [51].

It is worth noting that in addition to the direct effect of selenium on the biosynthesis of antioxidant compounds discussed above, selenium can indirectly affect their concentration in lamb’s lettuce due to effects on leaves nitrate metabolism. The previous study demonstrated the stimulation of nitrate metabolism and a reduced nitrate accumulation in the leaves of a lettuce species after selenium application [24]. Reduced nitrate accumulation can in turn lead to increased antioxidant levels in lamb’s lettuce, as it was shown in the study [12]. However, further study on the effect of selenium application on nitrogen metabolism in lamb’s lettuce is needed for more accurate identification of the reasons for the stimulating effect of selenium on the antioxidant level in lamb’s lettuce.

### 3.3. Changes in the Content of Hydrophilic and Lipophilic Antioxidants during Plant Growth

The current study of changes in the content of phytochemicals in lamb’s lettuce shoots during plant growth showed that the content of phenolic compounds decreased, and the content of chlorophylls and β-carotene increased by the last stage of harvesting (66 DAS) (Appendix A, Table A1). The higher level of phenolic compounds in young plants may be due to several reasons. First, young plants are generally more metabolically active, and second, in the early stages of growth, they require more compounds with protective including antioxidant properties [9]. The decrease in the content of phenolic compounds in more mature plants was most likely due to their dilution as the biomass of the plant increased [52].

The result obtained for ascorbic acid, namely the absence of significant changes in its content during plant growth, was somewhat unexpected. Previously, some studies have shown that the content of ascorbic acid in leaves also decreases as plants grow. In particular, such a result was obtained for purslane [9] and dill [53]. In studies on the effect of the harvest season and the growth stage on the vitamin C content in lamb’s lettuce grown in the field, it was shown that when harvesting lamb’s lettuce in early summer, young plants contained 54–57% more vitamin C than older plants. However, when harvesting plants at the end of summer, the vitamin C content in young plants was 9% lower than in older plants [54]. Thus, the patterns of changes in the content of ascorbic acid with the growth of the plant strongly depend on both the type of plant and the conditions of its growth.

Mature lamb’s lettuce plants harvested at 66 DAS accumulated more chlorophylls and β-carotene than immature or “young” plants harvested at 38 DAS. The maximum lutein content was reached at an earlier stage (52 DAS). A similar trend was observed in studies on the changes in the pigment content in kale [55], dill [53], and lettuce [6]. The distribution of carotenoids between the two photosystems is uneven. The pigments of photosystem I (PS I) are enriched with β-carotene, while lutein dominates in photosystem II. PS II develops in leaf tissues earlier than PS I, which leads to a maximum increase in the lutein concentration at an earlier stage compared to other pigments [55].

The maximum antioxidant activity of hydrophilic extracts was detected at the first stage of harvesting, which corresponded to changes in the contents of hydroxycinnamic acids and total phenolic compounds during plant growth and is explained by a high correlation between these parameters (Table 2; Appendix A, Table A1). The antioxidant activity of lipophilic extracts, on the contrary, at the first stage of harvesting (38 DAS) was minimal and correlated with changes in the contents of lutein and total carotenoids (Table 3; Appendix A, Table A2).

The conducted studies were supposed to make it possible to determine the optimal time of harvesting lab’s lettuce to obtain products with high medicinal and nutritional value. However, the obtained result indicates the ambiguity of such a choice. Thus, if the goal is to obtain a product with a higher content of hydrophilic antioxidants, then preference should be given to young plants. If it is planned to use the lamb’s lettuce as a raw material, for example, to obtain biologically active additives with a high content of lutein and β-carotene, it is better to harvest more mature plants.

## 4. Materials and Methods

### 4.1. Plant Materials

The experiment was conducted in a greenhouse at Immanuel Kant Baltic Federal University from 10 December 2020 to 20 March 2021. Lamb’s lettuce seeds (*Valeriana locusta* L. Laterr. cv. Expromt) were purchased from Johnsons^TM^ (Kentford, UK). Seeds were sown in perlite and watered with a nutrient solution diluted 2-fold. Fourteen-day-old plants of the same size were transferred to 5-liter pots with a nutrient solution diluted 2-fold. Four plants were planted in each pot. After a 7-day adaptation period, the plants were used for the experiment. Selenium was added to the nutrient solution in the form of sodium selenate. Three selenium levels were studied (5.0, 10.0, and 20.0 µM). Plants grown on a nutrient solution without the addition of selenium were used as a control. The nutrient solutions consisted of the following macroelements: 4.75 mM N-NO_3_^−^, 0.25 mM N-NH_4_^+^, 1 mM P-H_2_PO_4_^−^, 2 mM K^+^, 3 mM Ca^2+^, 1.5 mM Mg^2+^, 2 mM S-SO_4_^2−^; and microelements: 35 µM Fe^2+^, 7 µM Mn^2+^, 20 µM B-BO_3_^3−^, 0.7 µM Cu^2+^, 0.5 µM Mo-MoO_4_^2−^, 7 µM Zn^2+^. The nutrient solutions were maintained at a pH of 5.6 ± 0.1, an electrical conductivity (EC) of 2.1 ± 0.1 dS m^−1^, and were renewed weekly.

The average temperature in the greenhouse during the experiment was 17.9 °C and the average relative humidity was 66%. The photoperiod duration was 16 h at an illumination intensity of 240 µmol/m^2^ s.

Lamb’s lettuce was harvested at three growth stages (38, 52, and 66 days after sowing (DAS)). This corresponded to 14, 28, and 42 days of selenium treatment of the plants. At the last stage (66 DAS), the lamb’s lettuce was at the stage of commercial maturity.

Four replications for each treatment (Se concentration and growth stage) were conducted. There were 48 pots in total. The pots were arranged in a completely randomized design.

After harvesting, the fresh weight of plant shoots was determined. To determine the dry weight, water content, and selenium concentration, part of the plant material was dried at 60 °C for 72 h. The other part of the plants was frozen in liquid nitrogen, lyophilized, crushed, and used for the extraction of phenolic compounds, chlorophylls, and carotenoids. The determination of vitamin C was carried out on the day of the harvesting in fresh material. The plants from each pot were mixed and represented an average sample of four plants.

### 4.2. Determination of Se Concentration

The Se concentration was measured by hydride generation atomic absorption spectrometry HG-AAS (SpectrAA 220 FS with VGA 77 vapor generation accessory, Agilent Inc., Santa Clara, CA, USA) as described in [56]. Oven-dried ground shoots were mineralized by high-pressure autoclave decomposition. In total, 0.5 g of dried plant samples was put into a fluoroplastic beaker (“closed vessel”), then HNO_3_ (concentrated) was added and 10 min later, H_2_O_2_ (30% water solution) was added into the reaction solution. The fluoroplastic beaker was then hermetically sealed and put into an oven. The mineralization of the sample was carried out at a temperature of 180 °C for 2 h. After the beaker got cold, its content was poured into a colorimetric test tube and a concentrated HCl and an amidosulfonic acid solution were added. Thereafter, the content of the test tube was kept at a water bath at a temperature of 70 °C for 1 h. The obtained solution was used for HG-AAS analysis [52]. Blank solution contained all chemicals without plant material or Se standard and passed all stages of analysis. The samples were analyzed in three replicates. The Se concentration was expressed as μg per gram dry weight of plant material.

### 4.3. Determination of Vitamin C

To determine vitamin C, fresh plant material was homogenized in a 5% (*w/v*) aqueous solution of meta-phosphoric acid containing 1% (*w/v*) dithiothreitol according to [57]. The vitamin C concentration was estimated using the AOAC (Association of Official Analytical Chemists) method as described in [58]. Plant homogenate was titrated with 0.05% 2,6-dichlorophenol-indophenol solution in sodium bicarbonate, until the presence of a rosy color for more than 5 s. The indophenol solution was standardized daily with the L-ascorbic acid solution. The determinations were repeated three times.

### 4.4. Determination of Phenolic Compounds

Phenolic compounds were extracted from lyophilized crushed plant material. Extraction was carried out three times with a 60% (*v/v*) aqueous methanol solution [59]. The resulting combined extract was filtered and used to determine the total content of hydroxycinnamic acids, the total content of flavonoids, the total content of phenolic compounds, and for high-performance liquid chromatography (HPLC) analysis of individual phenolic compounds.

The total content of hydroxycinnamic acids was determined spectrophotometrically using the Arno’s reagent consisting of sodium nitrite and sodium molybdate [60]. Standard solutions of chlorogenic acid were used for the calibration curve. The total content of flavonoids was determined spectrophotometrically using aluminum chloride [61]. Quercetin was used as a standard. The total content of phenolic compounds was determined using the Folin–Ciocalteu reagent [62]. Gallic acid was used as a standard. Spectrophotometric studies were carried out on a Shimadzu UV-3600 spectrophotometer (Shimadzu, Kyoto, Japan).

**Figure A1:**
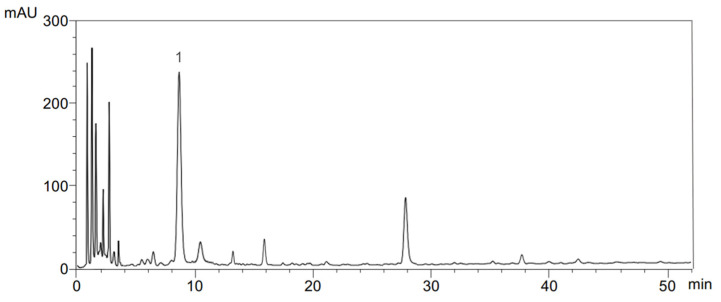
Exemplary HPLC chromatogram of the phenolic acids and flavonoids in lamb’s lettuce at 320 nm: 1—chlorogenic acid.

HPLC with diode-array detection (HPLC-DAD) was used to identify and quantify individual phenolic compounds (Shimadzu LC-20 Prominence chromatograph, Shimadzu, Kyoto, Japan). Phenolic compounds were separated using the Phenomenex Luna column (C18, length: 250 mm, diameter: 4.6 mm, particle size: 5 µm) and gradient elution mode. A mixture of solvents—water/trifluoroacetic acid (*v/v*) 99.0/1.0 (A) and acetonitrile (B)—was used as the mobile phase. The flow rate was 0.85 mL/min, the column temperature was 40 °C, and the sample volume was 20 µL. Detection was carried out in the range of 180–750 nm. The compounds were identified by the retention time of the peaks obtained on the chromatograms and their comparison with the retention times of the standards. Quantitative analysis of phenolic compounds was carried out using calibration curves created in the concentration range of 1–100 µg/mL. The following substances were used as standards: chlorogenic acid, rosmarinic acid, 3,4-dihydroxybenzoic acid, caftaric acid, caffeic acid, chicoric acid, hyperoside, luteolin-7-glucoside, rutin, quercetin-3-O-glycoside, apigenin-7-O-glucoside, and kaempferol-3-glucoside (Merck Life Science LLC, Moscow, Russia). An exemplary HPLC chromatogram of the phenolic acids and flavonoids in lamb’s lettuce is presented in Appendix B on Figure A1.

### 4.5. Determination of Chlorophylls and Carotenoids

Chlorophylls and carotenoids were extracted from lyophilized crushed plant material using a 1:1 solution of methanol and tetrahydrofuran as a solvent. Extraction was repeated until the plant sample was discolored. The combined extract was dried under nitrogen. The pigment precipitate was dissolved in a mixture of dichloromethane and isopropanol (ratio 1:4) and filtered out [63]. The resulting extract was used for chromatographic determination of chlorophylls and carotenoids.

**Figure A2:**
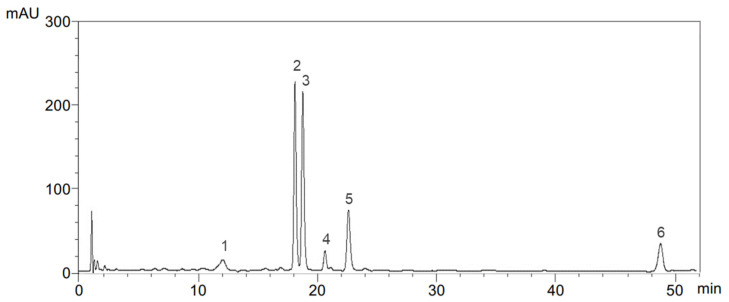
Exemplary HPLC chromatogram of the chlorophylls and carotenoids in lamb’s lettuce at 450 nm: 1—neoxanthin, 2—chlorophyll *b*, 3—lutein, 4—zeaxanthin, 5—chlorophyll *a*, 6—β-carotene.

The HPLC-DAD method was used to separate, identify, and quantify the content of chlorophylls and carotenoids (Shimadzu LC-20 Prominence chromatograph, Shimadzu, Kyoto, Japan). The pigments were separated using the Phenomenex Luna column (C18, length: 250 mm, diameter: 4.6 mm, particle size: 5 µm) and gradient elution mode. As components of the mobile phase, a mixture of solvents was used: methanol/*tert*-butyl methyl ether/water (81/15/4, *v/v*) containing 20 mM ammonium acetate (A) and *tert*-butyl methyl ether/methanol/water (90/6/4, *v/v*) containing 20 mM ammonium acetate (B). The flow rate was 1.0 mL/min, the column temperature was 25 °C, and the sample volume was 10 µL. Absorption spectra were recorded in the range of 190–750 nm. The identification of pigments was carried out by comparing the absorption spectra and retention time obtained on the chromatograms of peaks, by comparing them with the corresponding parameters of standards, or by comparing the absorption spectra reported in the literature. Quantitative determination was carried out using calibration curves. The following substances were used as standards: chlorophyll *a*, chlorophyll *b*, lutein, and β-carotene (Merck Life Science LLC, Moscow, Russia). The total content of chlorophylls was calculated as the sum of the contents of chlorophyll *a* and chlorophyll *b*. When calculating the total content of carotenoids, the areas of the peaks of carotenoids for which there were no standards were used to calculate the quantitative content of carotenoids in terms of lutein and were summed with the contents of lutein and β-carotene. An exemplary HPLC chromatogram of the chlorophylls and carotenoids in lamb’s lettuce is presented in Appendix B on Figure A2.

### 4.6. Determination of the Antioxidant Activity of Hydrophilic and Lipophilic Extracts

To investigate the difference in the antioxidants activity of lipophilic and hydrophilic plant components, two different types of extraction procedures were used. The “hydrophilic” extracts were prepared as described for phenolic compound extraction and the “lipophilic” extracts were prepared as described for chlorophylls and carotenoids extraction (see above).

The antioxidant activity of hydrophilic and lipophilic extracts was determined using ABTS^+•^ (2,2’-azino-bis(3-ethylbenzothiazoline-6-sulfonic acid) radical according to the Trolox equivalent antioxidant capacity (TEAC) assay and ferric-reducing antioxidant power (FRAP) assay as described previously by the authors [64]. Trolox was used as a standard in both assays.

The content of all the studied phytochemicals was expressed in mg per gram dry weight. Antioxidant activity was expressed in µmol Trolox equivalent per gram dry weight.

### 4.7. Statistical Analysis

The experimental data were processed statistically using SigmaPlot 12.3 program (Systat Software GmbH, Erkrath, Germany). Statistical analysis of data was performed only with biological replications corresponding to the number of pots in each treatment (*n* = 4). The results in the graphs and tables are presented as a mean value ± standard deviation (*n* = 4). To assess the significance of the differences between the treatments, one-way ANOVA was performed, followed by the use of the Tukey test with significance at *p* ≤ 0.05. Two-way ANOVA was used to assess the influence of factors (Se concentration, growth stage at harvest) and the effect of their interaction. Correlation analysis was carried out using Pearson correlation analysis.

## 5. Conclusions

This study of the effect of various selenium concentrations showed that the addition of 5 µM of selenium to the nutrient solution stimulated plant growth, and also led to higher contents of chlorogenic acid, total hydroxycinnamic acids, flavonoids, phenolic compounds, ascorbic acid, and antioxidant activity of hydrophilic lamb’s lettuce extracts compared with untreated plants. In addition, this concentration of selenium provided a high but at the same time safe for human health concentration of selenium in the plant. This study of changes in the content of hydrophilic and lipophilic antioxidants during plant growth showed that the maximum content of hydrophilic antioxidants was in young plants, and the maximum content of lipophilic antioxidants was in more mature plants. Thus, the obtained results confirm the possibility of selenium biofortification of lamb’s lettuce, which will not only increase the level of this trace element in the human diet but also allow plants with greater nutritional, including antioxidant, value to be obtained.

## Figures and Tables

**Table 1:** Effect of Se concentration in nutrient solution on biomass and selenium accumulation in lamb’s lettuce harvested at different growth stages.

Growth Stage at Harvest	Se Concentration, µM	Se Concentration µg g^−1^	Se Content µg Plant^−1^	FW Shoots g Plant^−1^	DW Shoots g Plant^−1^	Dry Matter Content %
38 DAS	0	0.051 f ^1^	0.009 f	2.21 e	0.186 d	8.42 a
	5	2.11 ef	0.388 f	2.19 e	0.186 d	8.51 a
	10	11.58 d	2.27 ef	2.23 e	0.195 d	8.78 a
	20	39.29 c	6.86 de	2.16 e	0.175 d	8.17 a
52 DAS	0	0.018 f	0.009 f	6.64 d	0.533 c	8.03 a
	5	6.29 def	3.71 ef	7.18 d	0.591 c	8.23 a
	10	39.3 c	21.6 c	6.77 d	0.550 c	8.13 a
	20	124.4 a	65.1 a	6.58 d	0.524 c	7.97 a
66 DAS	0	0.011 f	0.010 f	11.62 b	0.899 b	7.76 a
	5	10.7 de	12.28 d	14.72 a	1.201 a	8.14 a
	10	37.2 c	31.5 b	10.99 bc	0.850 b	7.73 a
	20	90.8 b	68.9 a	9.77 c	0.763 b	7.80 a
**Main effects ^2^**						
Se concentration (Se)	0 (control)	0.0264 d	0.009 d	6.82 b	0.539 b	8.07 a
	5 µM	6.22 c	5.46 c	8.0. a	0.660 a	8.30 a
	10 µM	29.34 b	18.48 b	6.66 bc	0.531 b	8.21 a
	20 µM	84.83 a	46.98 a	6.17 c	0.488 b	7.98 a
Harvest (H)	38 DAS	13.26 c	2.38 c	2.20 c	0.186 c	8.47 a
	52 DAS	42.52 a	22.63 b	6.79 b	0.550 b	8.09 ab
	66 DAS	34.58 b	28.20 a	11.78 a	0.929 a	7.86 b
Significance	Se	<0.001 *	<0.001 *	<0.001 *	<0.001 *	0.424 ^ns^
	H	<0.001 *	<0.001 *	<0.001 *	<0.001 *	0.005 *
	Se * H	<0.001 *	<0.001 *	<0.001 *	<0.001 *	0.850 ^ns^

^1^ Data was evaluated via one-way ANOVA followed by the Tukey HSD test (mean, *n* = 4). ^2^ Data was evaluated via two-way ANOVA, factors: selenium concentration in nutrient solution and growth stage at harvest, followed by the Tukey HSD test (mean, *n* = 4). Identical letters indicate that values do not differ significantly. Asterisks (*) indicate significantly influential factors, ns, not significant. FW, fresh weight, DW, dry weight.

**Table 2:** Correlation matrix with the Pearson coefficient values for the ascorbic acid, phenolic compounds, and antioxidant activity of hydrophilic extracts.

Parameters	THA ^1^	TFC	TPC	AsA	TEAC-H	FRAP-H
ChlA	0.99 **	0.52 *	0.96 **	0.06 ^ns^	0.80 **	0.75 **
THA		0.54 *	0.97 **	0.03 ^ns^	0.79 **	0.74 **
TFC			0.68 **	0.35 *	0.47 *	0.52 *
TPC				0.08 ^ns^	0.79 **	0.76 **
AsA					0.19 ^ns^	0.47 *
TEAC-H						0.85 **

^1^ THA, total hydroxycinnamic acids; TFC, total flavonoids content; TPC, total phenolics content; TEAC-H, Trolox equivalent antioxidant capacity of hydrophilic extracts, FRAP-H, ferric-reducing antioxidant power of hydrophilic extracts; ChlA, chlorogenic acid. ** Correlation is significant at *p* ≤ 0.01; * correlation is significant at *p* ≤ 0.05; ns, correlation is not significant (*p* > 0.05).

**Table 3:** Correlation matrix with the Pearson coefficient values for the chlorophylls, carotenoids, and antioxidant activity of lipophilic extracts.

Parameters	Chl *b* ^1^	TChl	Lut	β-Car	TCar	TEAC-L	FRAP-L
Chl *a*	0.56 *	0.98 **	0.51 *	0.64 **	0.66 **	0.82 **	0.30 *
Chl *b*		0.67 **	−0.04 ^ns^	0.70 **	0.10 ^ns^	0.35 *	−0.18 ^ns^
TChl			0.45 *	0.69 **	0.61 **	0.80 **	0.23 ^ns^
Lut				0.42 *	0.92 **	0.74 **	0.44 *
β-Car					0.57 *	0.56 *	−0.11 ^ns^
TCar						0.80 **	0.35 *
TEAC-L							0.48 *

^1^ Chl *b,* chlorophyll *b*; TChl, total chlorophylls; Lut, lutein; β-Car, β-Carotene; TCar, total carotenoids; TEAC-L, Trolox equivalent antioxidant capacity of lipophilic extracts, FRAP-L, ferric reducing antioxidant power of lipophilic extracts; Chl *a,* chlorophyll *a*. ** Correlation is significant at *p* ≤ 0.01; * correlation is significant at *p* ≤ 0.05; ns, correlation is not significant (*p* > 0.05).

## Data Availability

The data presented in this study are available on request from the corresponding author.

## Appendix A

**Table A1:** Results of two-way ANOVA for the phenolic compound content in lamb’s lettuce and antioxidant activity of hydrophilic extracts.

Factors	Level	Chlorogenic Acid mg g^−1^	THA mg g^−1^	TFC mg g^−1^	TPC mg g^−1^	AsA mg g^−1^	TEAC -H µmol g^−1^	FRAP-H µmol g^−1^
Main effects ^1^								
Se concentration (Se)	0 (control)	5.907 c	6.358 c	1.386 c	10.353 c	5.284 c	74.293 b	132.102 c
	5 µM	7.499 a	8.034 a	1.579 b	12.946 a	6.845 b	99.434 a	172.775 a
	10 µM	6.295 b	6.796 b	1.626 ab	11.228 b	6.859 b	75.601 b	149.857 b
	20 µM	5.667 c	6.007 c	1.691 a	10.221 c	7.309 a	74.910 b	146.069 b
Harvest (H)	38 DAS	6.964 a	7.220 a	1.808 a	12.797 a	6.401 a	88.887 a	158.935 a
	52 DAS	6.606 b	6.483 b	1.591 b	11.495 b	6.727 a	81.971 b	150.513 b
	66 DAS	5.456 c	5.370 c	1.313 c	9.269 c	6.595 a	72.320 c	141.069 c
Significance	Se	<0.001 *	<0.001 *	<0.001 *	<0.001 *	<0.001 *	<0.001 *	<0.001 *
	H	<0.001 *	<0.001 *	<0.001 *	<0.001 *	0.072 ^ns^	<0.001 *	<0.001 *
	Se * H	0.078 ^ns^	0.079 ^ns^	0.114 ^ns^	0.011 *	0.009 *	<0.001 *	0.006 *

^1^ Data was evaluated via two-way ANOVA, factors: selenium concentration in nutrient solution and growth stage at harvest, followed by Tukey HSD test (mean, *n* = 4). Identical letters indicate that values do not differ significantly. Asterisks (*) indicate significantly influential factors, ns, not significant. THA, total hydroxycinnamic acids content, TFC, total flavonoids content, TPC, total phenolics content, AsA, ascorbic acid, TEAC-H, Trolox equivalent antioxidant capacity of hydrophilic extracts, FRAP-H, ferric reducing antioxidant power of hydrophilic extracts, DAS, days after sowing.

**Table A2:** Results of two-way ANOVA for the content of chlorophylls and carotenoids in lamb’s lettuce and the antioxidant activity of lipophilic extracts.

Factors	Level	Chlorophyll *a* mg g^−1^	Chlorophyll *b* mg g^−1^	Total Chlorophyllsmg g^−1^	Luteinmg g^−1^	β-Carotenemg g^−1^	Total carotenoidsmg g^−1^	TEAC-L µmol g^−1^	FRAP-L µmol g^−1^
Main effects ^1^									
Se concentration (Se)	0 (control)	11.300 ab	2.632 b	13.932 b	0.879 a	0.108 ab	1.082 a	83.829 a	58.987 b
	5 µM	11.923 a	2.944 a	14.867 a	0.740 b	0.0999 c	0.908 b	81.883 a	65.559 a
	10 µM	10.794 b	2.849 a	13.643 b	0.833 a	0.112 a	1.037 a	85.036 a	65.054 a
	20 µM	11.550 a	2.761 ab	14.311 ab	0.702 b	0.101 bc	0.949 b	80.246 a	61.300 ab
Harvest (H)	38 DAS	8.405 c	2.514 b	10.919 c	0.678 c	0.0908 b	0.819 c	69.594 b	59.402 b
	52 DAS	12.620 b	2.536 b	15.157 b	0.902 a	0.103 b	1.333 a	91.070 a	70.181 a
	66 DAS	13.151 a	3.339 a	16.490 a	0.787 b	0.123 a	1.030 b	87.582 a	58.592 b
Significance	Se	<0.001 *	0.003 *	0.001 *	<0.001 *	<0.001 *	<0.001 *	0.126 ^ns^	<0.001 *
	H	<0.001 *	<0.001 *	<0.001 *	<0.001 *	<0.001 *	<0.001 *	<0.001 *	<0.001 *
	Se * H	0.098 ^ns^	0.268 ^ns^	0.131 ^ns^	<0.001 *	0.193 ^ns^	0.031 *	0.015 *	0.001 *

^1^ Data was evaluated via two-way ANOVA, factors: selenium concentration in nutrient solution and growth stage at harvest, followed by Tukey HSD test (mean, *n* = 4). Identical letters indicate that values do not differ significantly. Asterisks (*) indicate significantly influential factors, ns, not significant. TEAC-L, Trolox equivalent antioxidant capacity of lipophilic extracts, FRAP-L, ferric reducing antioxidant power of lipophilic extracts, DAS, days after sowing.
